# A Scoping Review of Trauma Treatments among Legally Involved Adolescents with Learning, Cognitive, or Intellectual Disabilities: Identifying Clinically Relevant Research Gaps in the Literature

**DOI:** 10.1007/s40653-025-00742-w

**Published:** 2025-08-18

**Authors:** Jeanne McPhee, Cynthia Valencia-Ayala, Hannah State, Melissa Morales, Marina Tolou-Shams, Johanna B. Folk

**Affiliations:** 1 Department of Psychology and Behavioral Sciences, University of California, San Francisco, 1001 Potrero Avenue, Building 5, Suite 7M, San Francisco, CA 94110 USA; 2 The University of Chicago Law School, Chicago, IL USA; 3 University of California Santa Cruz, Santa Cruz, CA USA

**Keywords:** Trauma treatment, Juvenile justice, Learning disorders, Intellectual disability, Scoping review

## Abstract

Youth involved in the legal system (YILS) experience higher rates of posttraumatic stress and learning, cognitive, or intellectual disabilities (LCID) than their peers without legal involvement. Trauma-focused treatments often require significant cognitive skills that may present difficulties for youth with LCID; this mismatch could impact the effectiveness of treatment with this subgroup of youth. Although evidence-based trauma treatments exist for YILS and for youth with LCID, there is no published synthesis examining such interventions for youth with LCID who are also legally involved. The current scoping review synthesizes existing literature related to trauma treatments for this population. A search of seven literature databases and backward citation searching using previously published articles on trauma treatments for youth yielded 406 articles, four of which examined the effectiveness of trauma treatments for YILS with LCID. None of the included articles provided specific analyses for YILS with LCID, leading to continued questions about the effectiveness of trauma treatment for this subset of youth. Results demonstrate a paucity of research on trauma-focused treatments for YILS with LCID, which is concerning given the significant cognitive skills required to effectively participate in evidence-based trauma treatments. Specific treatment considerations and adaptations are provided as opportunity for future clinical and empirical work in this area.

**Supplementary Information:**

The online version contains supplementary material available at 10.1007/s40653-025-00742-w.

## Background

High rates of trauma exposure are well-documented among youth involved in the juvenile legal system (YILS; Duron et al., 2022). As many as 90% of youth who have been arrested have a history of trauma exposure and many have experienced more than one type of trauma (average of six distinct trauma types; (Folk et al., 2022). For many YILS, trauma exposure predates legal system involvement (Ford et al., 2013) however, legal system processes can be retraumatizing to youth, exacerbating trauma-related psychopathology and leading to clinically significant posttraumatic stress disorder (PTSD) symptoms. Such experiences of trauma and the lasting related symptoms have been associated with physical and behavioral health problems (e.g., substance use, co-occurring mental health concerns), lower academic and vocational achievement compared to their non-legally involved peers, and poor legal outcomes (e.g., recidivism, probation failure) for YILS (Mendel, 2011).

In addition to the high rates of trauma, research has shown that learning, cognitive, or intellectual disabilities (LCID) are highly prevalent among YILS, especially when compared to their non-legally involved peers (Zhang et al., 2011). About one third of youth in long-term secure custody facilities (e.g., detention, ranches) have a learning disability (Development Service Group, 2017), between 28 and 43% of YILS have special education needs (Kim et al., 2021; Kvarfordt et al., 2005; Morris & Morris, 2006; Wang et al., 2005), and YILS have high rates of fetal alcohol syndrome disorders (e.g., Fast et al., 1999; Streissguth et al., 1996). This may be, in part, due to the relationship between learning disorders, school discipline, and legal involvement, as youth with learning-related difficulties are disproportionately disciplined at and referred for arrest in school (Rodriguez, 2013; Stone & Zibulsky, 2015; Sweeten, 2006). Beyond learning and neurocognitive disabilities, the rate of other cognitive psychopathology is much higher than that of non-legally involved youth: attention deficit hyperactivity disorder (ADHD) among YILS is at least four times that of the rate among all youth (Eme, 2008). Importantly, disorders such as ADHD and PTSD have strong associations for a variety of reasons, including their underlying neurobiological correlates and their specific deficits that may put youth at risk for developing further psychopathology (for a review, see Silverstein, 2023).

Research has identified several evidence-based treatments for PTSD used specifically with YILS that are effective in reducing trauma symptoms (Rhoden, 2019; Zettler, 2021), including Trauma-Focused Cognitive Behavioral Therapy (TF-CBT; Cohen et al., 2017), Prolonged Exposure for Adolescents (Foa et al., 2008), Trauma and Grief Component Therapy for Adolescents (Saltzman, 2017), and Trauma Affect Regulation: A Guide for Education and Therapy (TARGET; Ford, 2020). These treatments include central elements of exposure, done through imaginal engagement or by speaking or writing about experienced traumatic event(s) with the guidance of a therapist. When working with YILS, these treatments have been adapted to be specific and salient to the population; for example, including psychoeducation regarding structural and systemic influences on the presentation of trauma in youths’ lives (e.g., Johnson & Davis, 2020). In fact, there is a significant body of research demonstrating the positive effects of trauma-focused treatment on reducing PTSD symptoms for YILS; most specifically, in addition to several of the previously listed treatments for LCID (i.e., TF-CBT, EMDR), group trauma treatments (e.g., TARGET, Cognitive Behavioral Intervention for Trauma in Schools [CBITS], Seeking Safety) provided for YILS has demonstrated effectiveness in symptom reduction and can be cost effective treatments (Zettler, 2021).

Given the significant potential consequences to YILS with PTSD and the high rates of LCID in this population, it is important to understand the effectiveness of evidence-based interventions for young people involved in the legal system who also struggle with learning and cognitive difficulties to determine if adaptations to these treatments are needed. Research has noted that trauma treatment is effective, and importantly, not harmful, to youth with LCID. Although a number of evidence-based trauma treatments (e.g., Child-Parent Psychotherapy, Exposure Therapy, Trauma-Focused Cognitive Behavioral Therapy [TF-CBT], and Eye Movement Desensitization and Reprocessing [EMDR]) have all shown to reduce of PTSD symptoms in youth with intellectual and developmental disabilities (Keesler, 2020), the question remains how these treatments perform for YILS with LCID, a group of youth with higher education-related needs than their non-legally involved peers with LCID.

Despite the evidence of effective trauma treatments for YILS and the growing evidence of effectiveness for youth LCID, multiply marginalized youth with LCID and legal system involvement present with unique challenges and considerations that may have significant implications for the implementation and efficacy of trauma treatment. Most of these treatments involve components that require substantial language production and comprehension skills, including language-based exposure elements. Given that YILS experience disruptions in schooling due to increased risk for discipline-based school removal (i.e., suspensions, expulsions; Fabelo et al., 2011) and incarceration (Belkin, 2020; Grigorenko et al., 2015; National Council on Disability, 2015) these treatments may prove difficult for YILS to effectively participate. Further, they experience gaps special education services while incarcerated, increasing rates of recidivism and potentially disrupting behavioral, cognitive, and academic progress (Kim et al., 2021; Ochoa et al., 2021). Therefore, in order to better understand the effects of trauma treatments YILS with LCID, we conducted a scoping review of the literature.

## Method

### Search Strategy

A backwards citation search using recent reviews on evidence-based trauma treatments for YILS (Rhoden, 2019; Zettler, 2021) and trauma-specific treatments for youth with intellectual and developmental disabilities (Keesler, 2020) was conducted. The authors then conducted a search for additional articles for inclusion using PubMed, PsycINFO, Embase, Web of Science, Sociological Abstracts & Social Services Abstracts, PTSDPubs, and Google Scholar. Search terms were harvested from articles identified in the aforementioned reviews and tested with individual searches to determine relevance to the current review. Boolean logic was then applied to combine similar and related terms between the five concepts (e.g., “juvenile justice” OR “court-involved youth”) AND (trauma OR “posttraumatic stress”) AND (therapy OR treatment) AND (“learning disabilities” OR “developmental disabilities”). Detailed search strategies can be found in Supplemental Material A. Backwards citation searching was also completed on articles selected for full-text review.

**Fig. 1:**
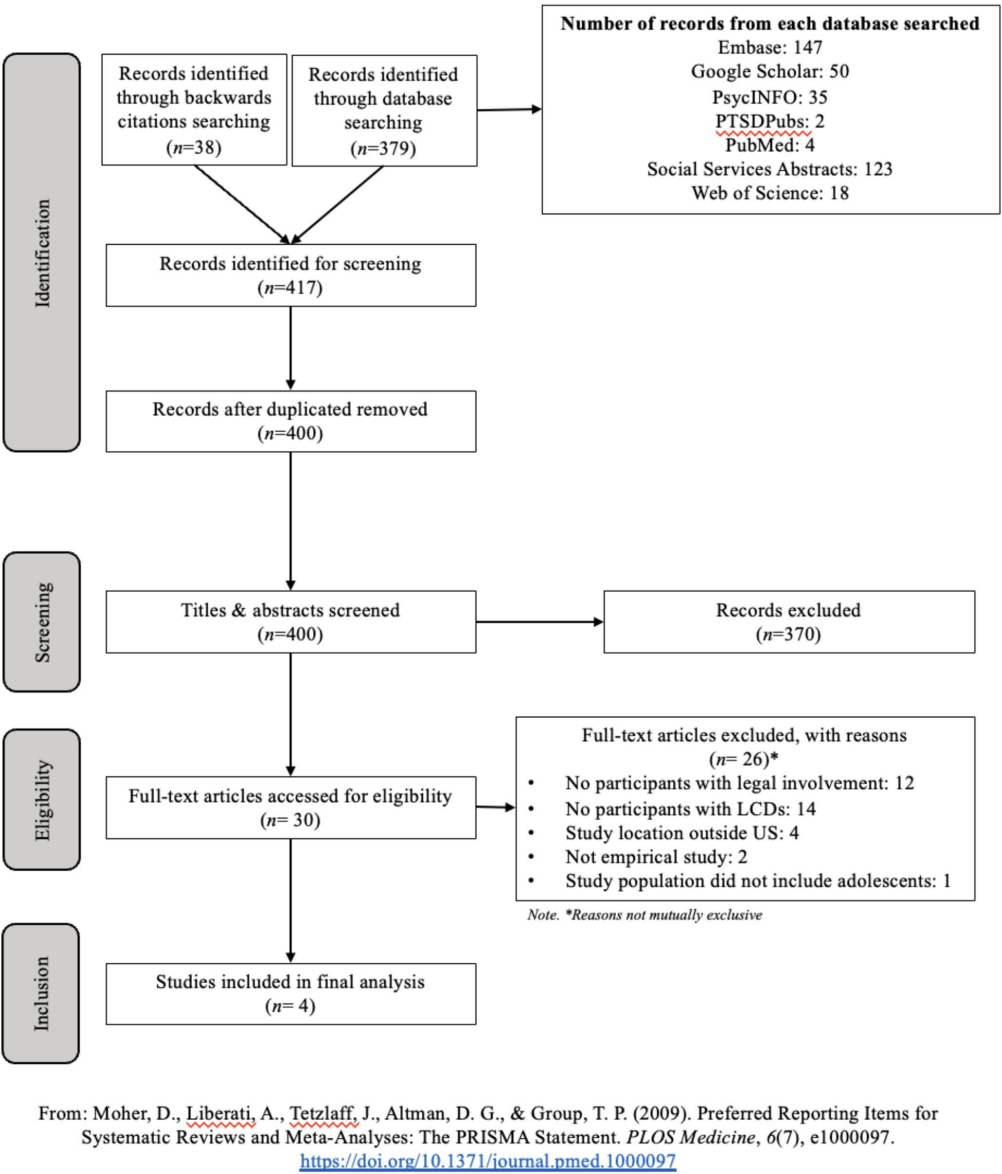
Flow chart of included studies

Our literature search yielded 417 articles (see Fig. 1 for PRISMA flow chart of the study screening process), 17 of which were identified as duplicates and excluded. Titles and abstracts of 400 articles were screened. Four reviewers screened titles and abstracts for a subset of articles (*n* = 20) to establish interrater reliability and the remaining articles were screened independently. To be eligible for inclusion in the full text review, articles had to present an empirical evaluation (e.g., pre-post-, randomized control trial, or quasi-experimental design) of a trauma-specific treatment for youth in the United States aged 10–18 years at the time of enrollment and published up until the search date (May 1, 2023). Trauma was not specifically defined in inclusion; thus, articles with any mention of trauma, posttraumatic stress, or adverse childhood experiences were moved to the full-text screening phase. Articles published in languages other than English, conceptual articles, literature reviews, or those focused on the medical treatment of physical injury such as dental or spinal trauma were excluded. The screening process revealed 30 articles that met criteria for full text review. Full-text articles screened for inclusion (*n* = 30) were then reviewed to ensure the papers included any participants who were legally involved at the time of study enrollment (e.g., arrested, court-involved, community-supervised, detained) and that any youth participants had documented learning, cognitive, or intellectual disability. Twenty-six of the articles did not meet study inclusion criteria [e.g., sample did not include adolescents (*n* = 1), YILS (*n* = 4), youth with any learning, cognitive, or intellectual disability (*n* = 6), or neither (*n* = 8); study took place outside of the United States (*n* = 4); article was not empirical in nature (*n* = 2); see Fig. 1 for more details]. The first author assessed and summarized findings from the four articles. Specific details extracted from each study included: treatment type, treatment modality (i.e., group, individual), treatment setting, number of sessions, length of sessions, participant demographics (i.e., gender, race, ethnicity, if provided), participant trauma history (if provided), participant LCID information (if provided), results of pre-post design on trauma symptoms (if provided), and results of analyses of youth with LCID vs. youth without LCID (if provided).

## Results

Four articles (13.3%) included YILS with LCID in their study sample. Sample sizes ranged from 2 to 66, with YILS with LCID or an associated condition (e.g., traumatic brain injury [TBI] with loss of consciousness) making up between 13 and 52% of each study sample. One study included YILS with documented special education status, and three included YILS with ADHD (one of which also included YILS with TBI with loss of consciousness). Of note, three of the four studies took place in a legal/custodial setting (e.g., detention).

Each included article examined the effects of a different treatment, though all used a cognitive behavioral orientation and included a component of narrative therapy (see Table 1). Treatments included Cognitive Processing Therapy, Eye Movement Desensitization and Reprocessing therapy, Trauma Affect Regulation: Guide for Education and Therapy (TARGET), and Cognitive Behavioral Intervention for Therapy in Schools Cultural Adaptation (CBITS-CA). While each article found effectiveness of trauma treatment for trauma and/or externalizing symptoms within their overall sample, none of the articles provided specific analyses for the subsample of YILS with LCID, thus making it impossible to understand the effects of trauma treatment for this specific group of youth. Given this, we summarize the articles independently and describe the samples and results of each.

**Table 1:** Characteristics of reviewed Full-Text studies on trauma treatment with YILS with LCD

	Sample Characteristics						Study Design		
	*N*	Age Range (M)	% Male	Race/Ethnicity*	% YILS	% with LCID	Study Design	Location	Trauma Assessment
Aherns & Rexford (2002)	38	15–18	100	60.5% White,	100	52% reported history of head injury with loss of consciousness,	Waitlist control vs. active treatment	Youth facility for adolescent offenders	Clinical interview with DSM-IV PTSD criteria,
		(16.4)		26.3% African American,		40% reported history of ADHD			PTSD Symptom Scale Self-Report (PSS-SR)
				5.3% Hispanic,					
				5.3% Native American,					
				2.6% Other race					
Ford et al. (2012)	59	13–17	0	59% Latina or mixed race,	100	13% met criteria for ADHD	RCT comparing TARGET with relational supportive therapy (ETAU)	Community	TESI-C/SR,
		(14.7)		25% White,					CAPS-CA,
				16% Black					Post-traumatic cognitions inventory, TSCC
Johnson & Davis (2020)	54	NR	72	37% White,	100	50% Special Education status	Cohorts based on housing unit randomly assigned to either CBITS-CA or business-as-usual control conditions	Juvenile Detention Center	UCLA PTSD-RI: DSM-V Version
		(NR)		22% Mixed race,					
				19% Hispanic,					
				13% African American,					
				4% Asian,					
				4% other race,					
				2% Native American					
Greenwald (2000)	2	15	100	NR	100	50% diagnosed with ADHD	Case Study	Open residential treatment program	None
		(15)							
	Study Design, cont.						Study Outcomes		
	Intervention		Modality		# Sessions		Results		
Aherns & Rexford	Cognitive Processing Therapy		Individual		8		Significant changes over time in reported symptomatology across active and waitlist control groups (*p* = .0001);		
(2002)							Treatment group scores on the PSS-SR fell by over 50% 4 weeks following treatment completion		
Ford et al. (2012)	Trauma Affect Regulation: Guide for education and Therapy (TARGET)		Individual		12		Youth in both treatment groups demonstrated clinically significant reductions in trauma symptoms (*p* < .001);		
							TARGET was twice as effective in reducing overall PTSD symptom severity (62%) compared to ETAU (35%)		
Johnson & Davis (2020)	CBITS with Cultural Adaptations (CBITS-CA)		Group		10–12;		No significant differences between treatment groups;		
					9 group with 1–3 individual		Youth in both groups showed significant decreases in behavioral health symptomatology following treatment;		
							Youth overall showed average reduction of 10 points on UCLA-RI		
Greenwald (2000)	EMDR-infused motivational interviewing, cognitive behavioral training, and trauma processing individual therapy program		Individual		5–15		Positive outcomes reported for both youth including fewer interpersonal conflicts, increased school engagement, improved affect regulation, and limited future legal involvement		

*Note. Race/ethnicity is presented how these variables were reported in the published manuscripts

### Cognitive Processing Therapy

Ahrens & Rexford (Ahrens & Rexford, 2002) sought to compare the effects of Cognitive Processing Therapy (CPT) to a waitlist control group for incarcerated adolescent males. CPT was initially developed as a treatment for adults with significant PTSD symptoms, though it has been found to be effective for use with adolescents (Vogel & Rosner, 2020). It consists of three major components: psychoeducation about PTSD, trauma, and symptomology; exposure to memories of and thoughts/beliefs about the trauma using a written narrative; and cognitive therapy in which clients identify thoughts and emotions and challenge maladaptive thoughts and beliefs related to power, safety, trust, intimacy, and self-esteem. Participants in the CPT treatment group received eight, 60-minute sessions using the CPT protocol outlined by Resick and Schnicke (1993).

Participants (*n* = 38) were between 15 and 18 years old and identified as 60.5% White, 26.3% African American, 5.3% Hispanic, 5.3% Native American, and 2.6% other. Clinicians conducted psychodiagnostic interviews and used the PTSD Symptom Scale Self-Report (Coffey et al., 1998) to assess trauma exposure and related symptomology prior to treatment and at 4-weeks post-treatment; collateral records were also obtained from hospitals and social service investigations to assess trauma histories. While all participants had been exposed to trauma in their lifetimes, 68% had documented trauma histories and 29% reported experiencing more than one trauma in their lifetime. Over half (52%) reported a head injury with loss of consciousness and 40% reported a diagnosis of Attention-Deficit Disorder (ADD) or ADHD. Results demonstrated a significant group by time effect, such that youth participating in CPT experienced a greater reduction in PTSD symptoms compared to youth in the waitlist control group. In the CPT group, PTSD scores fell by over 50% from the pre-to post-treatment timepoints. There were no specific analyses examining attrition or treatment effects for youth who reported a significant head injury or previous diagnosis of ADD/ADHD.

### Trauma Affect Regulation: Guide for Education and Therapy

Ford and colleagues (2012) conducted a randomized control trial to test the efficacy of Trauma Affect Regulation: Guide for Education and Therapy (TARGET) versus enhanced treatment as usual (ETAU) for girls with a history of delinquency in a community setting. TARGET is a 12-session, individual treatment in which youth are provided psychoeducation about PTSD and trauma and sequential skills for emotion regulation prior to creating a personalized life narrative that includes traumatic and stressful events. The enhanced treatment as usual in this study was a manualized intervention focused on goal setting and problem solving through a relational framework; no psychoeducation or specific emotion regulation skills were discussed in the ETAU intervention.

Fifty-nine adolescent (ages 13–17 years) girls participated in this study; over half identified as Latina or mixed race, one quarter (25%) as White, and 16% as Black. 13% of participating youth met diagnostic criteria for ADHD. All youth reported a history of delinquency and met full or partial criteria for PTSD, as screened by a clinician using the Clinician Administered PTSD Scale for Children and Adolescents (CAPS-CA; Newman, 2002). Youth also completed the Traumatic Events Screening Inventory–Child/Self-Report (TESI-C/SR; Ford et al., 2002) to report trauma exposure, the Trauma Symptom Checklist for Children (TSCC; Briere, 1996) to report trauma-specific symptoms, and the Post-traumatic Cognitions Inventory (Foa et al., 1999) that assesses posttraumatic beliefs related to the world. Assessments were completed prior to randomized group assignment and again approximately four months later.

Results demonstrated that youth in both the TARGET and ETAU groups reported a clinically significant reduction in trauma symptoms. There was a significant group by time interaction, such that youth receiving TARGET showed greater reduction of PTSD symptoms compared to those receiving ETAU. There were no reported analyses examining attrition or treatment effects specifically among youth who met criteria for ADHD.

### Motivation-Adaptive Skills-Trauma Resolution Treatment

Greenwald (2000) provided a two-subject case study of treating incarcerated adolescent (age 15 years) boys with a history of conduct disorder with a novel approach to therapy, later called Motivation-Adaptive Skills-Trauma Resolution (MASTR) Treatment. MASTR is comprised of three phases: Motivation, in which motivational interviewing is used to conduct treatment planning; Adaptive Skills, in which cognitive behavioral techniques are introduced to reduce distress; and Trauma Resolution, in which Eye Movement Desensitization and Reprocessing (EMDR) is used to decrease stress and increase trauma-related problem solving (Greenwald, 2000). EMDR therapy is exposure-based psychotherapy treatment for trauma in which clients focus on an external stimulus (bilateral eye movements most commonly) while attending to descriptions of the traumatic event experienced by the client as well as client’s emotions, bodily sensations, and beliefs related to the traumatic event.

No demographic information other than participant age and gender was reported. One participant had previously been diagnosed with ADHD and oppositional defiant disorder; the other had no reported LCID. No standardized measurement was completed upon treatment termination, however the authors reported positive outcomes for both youth, including fewer interpersonal conflicts, increased school engagement, improved affect regulation, and limited future legal involvement.

### Cognitive Behavioral Intervention for Trauma in Schools

Johnson & Davis (2020) evaluated the efficacy of the Cognitive Behavioral Intervention for Trauma in Schools (CBITS) with Cultural Adaptations (CBITS-CA), a CBITS intervention with specific session information on racial trauma, versus treatment as usual for youth. The evaluation was conducted at a juvenile detention facility in Washington. The CBITS is a CBT-based group and individual intervention aimed to reduce youths’ symptoms of PTSD and depression caused by exposure to violence. The CBITS involves 10 group sessions and two or three individual sessions for one-on-one processing of traumatic events with a group co-facilitator. Additionally, CBITS includes a two-session caregiver education program and a one-session teacher education program. CBITS has demonstrated effectiveness for diverse populations of youth (Jaycox et al., 2018). The CBITS-CA had several adaptations, including combining the group exposure sessions (Sessions 5 and 6) into one due to small group sizes and adapting Session 9 to be an “Introduction to Race and Links to Traumatic Stress” in which youth discussed “what racism is, how it is defined, hear from others’ experiences with racism (through a video), how racism contributes to traumatic stress, and how one may apply skills learned from CBITS-CA to cope and combat racism” (Johnson & Davis, 2020; p. 9). No parent or teacher sessions were conducted due to logistical barriers.

Seventy youth participated in the study; 63% identified as a person of color, with 22% identifying as mixed race, 19% as Hispanic, and 13% African American. 50% of participants had documented special education status (though no breakdown of special education classification was provided). Given the setting, all youth had a history of delinquency and most (70%) had a length of stay at the facility of less than or equal to six months at the time of their participation. Youth were assessed with the UCLA PTSD Reaction Index: DSM-V Version (Kaplow et al., 2020), and all youth endorsed a history of trauma and scored a two or higher on at least one item in each of the four measured symptom categories. Assessments were completed prior to treatment group assignment and again following the treatment.

Results demonstrated that youth in both the CBITS-CA and the TAU groups showed significant reductions in behavioral health symptomology following treatment. Specifically, youth showed a reduction by, on average, almost ten points on the UCLA PTSD Reaction Index; there was no significant difference between CBITS-CA and TAU groups on changes in PTSD symptoms. Youth who participated in the CBITS-CA group experienced significant improvements in emotional expression in the face of racism and discrimination. There were no reported analyses examining attrition or treatment effects for youth with reported special education status.

## Discussion

The current review aimed to synthesize existing literature examining the effectiveness of trauma-focused treatment for YILS with LCID, however found no published articles addressing this question. Although research has demonstrated high rates of LCID among youth in legal settings, few articles examining the effectiveness of trauma treatment for adolescents included the population of interest; those studies that did implement trauma treatment in settings with adjudicated youth did not provide information about the effectiveness or efficacy of trauma treatment specifically for YILS with LCID. Therefore, researchers are left with an open question as to whether the various treatments studied work for this specific population.

Previous clinical services research has often excluded participants with learning disabilities (e.g., as there has been concern that such disabilities can add significant complexity to the treatment process (Reinecke & Shirk, 2007). However, studies have demonstrated limited evidence suggesting adverse responses to trauma treatment for youth with LCID. Furthermore, research indicates that therapy with components similar to those found in the included studies are efficacious for individuals with LCID and YILS. Specifically, there is evidence supporting cognitive behavioral therapy, exposure, and narrative therapy in the reduction of mental health symptoms for YILS (Blakeley-Smith et al., 2021; Mayer et al., 2023) and those with LCID (Sukhodolsky & Ruchkin, 2006).

The current review highlights an important gap in the literature and a clinical concern: youth with significant experiences of trauma, due to either or both their involvement in the legal system and the experience with school or their LCID, may not be receiving empirically supported and effective services to treat distressing and life-interfering trauma symptoms.

### Considerations for Treatment

Results from the included studies demonstrated that interventions with elements of cognitive behavioral therapy, exposure, and narration reduce trauma symptoms for YILS, some of whom also had LCID. This is consistent with recent reviews demonstrating effectiveness of similar trauma treatments (e.g., EMDR, TF-CBT) for YILS (Zettler, 2021) and youth with LCID (Keesler, 2020). Given the specific needs of YILS with LCID, especially for those who may have LCID challenges related to the language or executive functioning necessary to complete trauma narration, further research must be done to assess the utility and effectiveness of evidence-based trauma treatments for YILS with LCID.

Previous research has indicated that adaptations of trauma treatment for youth with LCID and YILS should be carefully considered. In fact, a recent study by D’Amico and colleagues (2022) found that clinicians providing TF-CBT to youth with developmental and intellectual disabilities reported making significant adaptations to treatment, including changes to pacing and session length, content and structure, and involvement of caregivers. Given the overlap of these two populations and the complex, individualized needs of YILS with LCID, adaptations to trauma treatment for this subset of youth are encouraged; while there is no empirical research demonstrating specific adaptations for this population, adaptations (see Table 2) can be considered by considering the needs of YILS and youth with LCID, per previous research (i.e., Zettler, 2021; Keesler, 2020). For example, clinicians providing trauma treatment to YILS with LCID should recognize the increased importance of physical and emotional safety in youths’ participation in treatment. YILS with LCID may feel physically unsafe due to their current legal status (e.g., participating in court-mandated treatment) or setting (e.g., incarceration) as well as emotionally unsafe due to their intellectual or cognitive deficits (e.g., having limited understanding of treatment or treatment providers). An unaddressed feeling of a lack of safety can lead to what may look like treatment resistance (e.g., missed sessions, avoidance), however, clinicians can modify their session planning to include additional time for rapport building, open discussion about fears and concerns related to treatment and confidentiality, and consideration for involving additional support people (when and where feasible). We highlight several areas of considerations and adaptations for trauma treatment for YILS with LCID in Table 2 (note that this compilation of suggestions is not meant to be an exhaustive list). For example, YILS with LCID, especially those such as Dyslexia, Specific Learning Disabilities, may have particular challenges with psychoeducation- and trauma narration-related activities due to significant missed schooling or cognitive difficulties related to reading and writing. Therefore, clinicians should provide visual aids and diagrams to supplement written psychoeducational materials, review homework verbally and complete an example using verbal instructions during session to model expectations without relying solely on written instructions, and use an audio recording device for trauma narration/exposure elements so clients are not required to reread their narrative to engage in exposure.

**Table 2:** Trauma treatment components’ challenges and adaptations given YILS with LCID clinical presentation

Treatment Component	Challenge	Client Presentation	Treatment Adaptation
*Safety & Rapport Building*	Feeling physically unsafe in treatment setting	Increased hypervigilance	Involve support people whenever possible in treatment; Ensure privacy of therapy space and situate clients in non-distracting areas of privacy space Where possible, use spaces specific to therapy (e.g., mindfulness stress reduction rooms); Provide coping skills for hypervigilance early in treatment and prompt for practicing in session
	Feeling emotionally unsafe with treatment provider or during trauma treatment	Resistance to treatment, including attrition/missed sessions; Avoidance of session topics/increased “crisis of the week” needs	Spend additional time building rapport; Provide additional psychoeducation about trauma treatment using developmentally appropriate language, visual stimuli; Hold open discussion with client about concerns and fears (including those related to court/probation reporting and requirements)
*Written Materials & Spoken Session Content*	Difficulty reading psychoeducational materials, written homework assignments, trauma narration	Avoidance of homework, trauma narration	Provide visual aids and diagrams to supplement written psychoeducational materials; Verbally review homework assignments and complete example during session; Audio-record trauma narrative and replay recording for youth during sessions with exposure component
	Struggling with writing trauma narration	Avoidance of homework, trauma narration	Use creative methods of trauma narration (e.g., drawing, puppet show, timeline, audio/visual recording); Assist with writing trauma narration if youth is uninterested in creative method
	Difficulty speaking and organizing speech during session	Lack of oral expression in session; avoidance of trauma narration	Use creative methods of trauma narration (e.g., drawing, puppet show); Use assistive technology (e.g., text-to-speech synthesizers, dictation with translation services)
	Difficulty understanding clinician	Misunderstanding or not capturing intended information during session; avoidance of relistening to trauma narration or exposure content	Ask youth to read back written trauma narration; Using assistive devices to aid auditory difficulties
*Session Organization*	Difficulty with session length, level of content	Distractibility; Low frustration tolerance demonstrated in later parts of session; Avoidance of session material	Use visual session agenda; Prompt clients verbally to remind of topic, agenda Be flexible with session location; Offer regular breaks; Shorten sessions and/or shorten agendas for session topics; Use multimedia presentation of information (e.g., videos, music); Make sessions interactive/fun; Provide rewards/incentives for on-task behavior (e.g., game time at the end of the session)
	Disorganization in trauma memories	Avoidance, lack of detail provided in trauma narration	Use visual reminder (e.g., timeline) to help client orient to details to provide in narration
	Challenges with remembering specific details, emotions, thoughts in trauma processing	Avoidance, lack of detail provided in trauma narration	Help client with perspective taking during cognitive processing (e.g., how might another person feel in this situation? Given that you felt [emotion] during [other situation], how might you have felt?)

It is also crucial that trauma treatments used and adapted for use with YILS with LCID are developmentally appropriate and take into consideration the developmental needs and stages of the youth receiving treatment. Many YILS who have experienced trauma have been exposed to significant and repeated interpersonal traumatic events; their trauma can be better understood as complex trauma (Complex Trauma Treatment Network of the National Child Traumatic Stress Network, 2016), as it has wide-ranging long-term impact on their daily lives. Complex trauma is a distinct diagnosis in the DSM-5, however there is advocacy and debate about distinguishing Complex Trauma from PTSD in future editions of the DSM (National Center for PTSD, U.S. Department of Veterans Affairs, 2025). In order to provide appropriate and effective trauma treatment for YILS with LCID, it is important providers assess for and understand the range of trauma-related needs youth may present with, including diagnostic features such as complex trauma, prior to selecting, adapting, and implementing trauma treatment.

### Limitations and Future Research

As this review highlights, there is extremely limited research available from which to draw conclusions about the effectiveness, feasibility, and acceptability of trauma treatments for YILS with LCID; given this, there are several limitations to this study. For example, learning, cognitive, and intellectual disabilities are not homogenous in their presentations and in how trauma treatments may function for youth with various LCID. For example, ADHD, a common diagnosis for YILS, may or may not have overlapping features and symptoms with an autism spectrum disorder or an intellectual disability; therefore, we are limited in generalizing from the limited number of studies in which any LCID was present for YILS in the sample. In addition, neurocognitive disorders are often misdiagnosed (including under- and over-diagnosis) in youth (Aggarwal & Angus, 2015; Charlot et al., 2022; Merten et al., 2017) especially due to the overlap in symptomology among LCID and related disabilities. These concerns related to the appropriate diagnosis of LCID are more apparent in socio-demographically underrepresented and minoritized groups (Morgan et al., 2023), especially as youth of color are more often diagnosed with behavioral disorders that may be better explained by LCID or trauma (e.g., oppositional defiant disorder; Ergun et al., 2021). Recommendations for considerations of appropriate diagnosis of LCID in youth have been published, including taking an antiracist approach to diagnostic evaluation (Legha, 2025). Given these concerns related to diagnostic clarity, and because youth with LCID are heterogeneous in their treatment-related needs, future research should focus on evaluating treatments for YILS with specific, common diagnoses and presentations (e.g., TBI, ADHD, autism spectrum disorder, fetal alcohol syndrome) in order to ascertain their effectiveness and appropriateness for use with subpopulations of YILS.

Clinical services researchers evaluating trauma treatments should consider expanding measurement and utilizing rigorous analytical methods to improve the field’s understanding of trauma treatments for YILS with LCID. In order to capture a young person’s legal involvement and educational and neurocognitive diagnostic history, researchers should include specific assessment of historical diagnosis and ongoing learning difficulties. Investigators may consider adding diagnostic measures to capture specific LCID symptoms that may overlap with trauma-related disorders (e.g., Conners-3 for ADHD symptomology; Conners, 2008); educational and/or legal records may inform the implementation of the intervention by providing contextual information that would illuminate potential facilitators or barriers to effective treatment within this population of YILS. Given the inconsistencies in demographic reporting across published studies, we recommend researchers report at minimum specific LCID diagnostic information, special education status, and experience with the legal system (e.g., arrests, detentions).

Furthermore, research on trauma treatments for YILS with LCID has focused mostly on youth who are detained, incarcerated, or in residential placement. Given that most YILS are living in the community and there are many intervention points along the sequential intercept (Folk et al., 2021; Heilbrun et al., 2017), it is crucial that researchers diversify locations and samples of YILS at earlier points in the pipeline (e.g., while under community supervision, in diversion programs). Implementation of treatment and its effects on the efficacy and effectiveness will likely be different among community-based and incarcerated youth.

Finally, the dearth of literature on providing trauma treatment for this subpopulation of youth has led to little diversity in analytical strategies to determine treatment effectiveness. Although clinical trials can provide information on symptom change, more research should be done to assess potential modifiers of treatment effectiveness, including, but not limited to, youths’ specific LCID, legal involvement, and other factors that may affect their symptom reduction over time (e.g., caregiver support, psychological flexibility; (Castro et al., 2023; Schramm et al., 2020). Regardless of methodology, it is important researchers include specific analyses that demonstrate the effectiveness of treatment specifically for YILS with LCID. In addition to effectiveness research, it is important that researchers assess the feasibility and acceptability of trauma treatments for YILS with LCID.

## Conclusion

This review synthesized the literature on the effectiveness of trauma-focused treatments for youth at the intersection of legal involvement and LCID. Significant gaps across the literature leave numerous empirical and clinical questions about the feasibility, acceptability, implementation, and effectiveness of interventions for reducing symptoms of trauma- and stressor-related disorders for youth with significant trauma-related needs. Therefore, we urge clinical services researchers to gather data related to legal involvement and educational and cognitive needs of youth and provide information related to the successes and challenges of treatment with this subgroup of youth in future published research. Finally, we encourage clinicians to adapt and individualize evidence-based treatments for clients with legal and cognitive and educational needs to ensure this clientele can maximally benefit from treatment.

## Supplementary Information

Below is the link to the electronic supplementary material.

Supplementary Material 1

## Data Availability

Not applicable, however, search strategy is provided in Supplemental Materials.

## References

[CR1] Aggarwal, S., & Angus, B. (2015). Misdiagnosis versus missed diagnosis: Diagnosing autism spectrum disorder in adolescents. *Australasian Psychiatry*, *23*(2), 120–123. 10.1177/1039856214568214 25653302 10.1177/1039856214568214

[CR2] Ahrens, J., & Rexford, L. (2002). Cognitive processing therapy for incarcerated adolescents with PTSD. *Journal of Aggression Maltreatment & Trauma*, *6*(1), 201–216. 10.1300/J146v06n01_10

[CR3] Belkin, L. D. (2020). Challenges with school re-entry for incarcerated youth and inadequacies of collaborative service provision by schools and agencies. *Handbook on promoting social justice in education* (pp. 2487–2523). Springer.

[CR4] Blakeley-Smith, A., Meyer, A. T., Boles, R. E., & Reaven, J. (2021). Group cognitive behavioural treatment for anxiety in autistic adolescents with intellectual disability: A pilot and feasibility study. *Journal of Applied Research in Intellectual Disabilities*, *34*(3), 777–788. 10.1111/jar.12854 33410240 10.1111/jar.12854

[CR5] Briere, J. (1996). Trauma symptom checklist for children (TSCC). *Journal of Abnormal PsychologyAssessment of Family Violence: A Handbook for Researchers and Practitioners*. 10.1037/t06631-000

[CR6] Castro, E., Magalhães, E., & del Valle, J. F. (2023). A systematic review of non-specific and specific treatment factors associated with lower or greater internalising and externalising symptoms in therapeutic residential care. *Children and Youth Services Review*, *147*, 106840. 10.1016/j.childyouth.2023.106840

[CR7] Charlot, L. R., Hodge, S. M., Holland, A. L., & Frazier, J. A. (2022). Psychiatric diagnostic dilemmas among people with intellectual and developmental disabilities. *Journal of Intellectual Disability Research*, *66*(10), 805–816. 10.1111/jir.12972 35974452 10.1111/jir.12972

[CR59] Choi, K. R., McCreary, M., Ford, J. D., Rahmanian Koushkaki, S., Kenan, K. N., & Zima, B. T. (2019). Validation of the traumatic events screening inventory for ACEs. *Pediatrics*, *143*(4), e20182546. 10.1542/peds.2018-2546 10.1542/peds.2018-2546 30837293

[CR8] Coffey, S. F., Dansky, B. S., Falsetti, S. A., Saladin, M. E., & Brady, K. T. (1998). Screening for PTSD in a substance abuse sample: Psychometric properties of a modified version of the PTSD symptom scale self-report. *Journal of Traumatic Stress*, *11*(2), 393–399. 10.1023/A:1024467507565 9565924 10.1023/A:1024467507565

[CR9] Cohen, J. A., Mannarino, A. P., & Deblinger, E. (2017). *Treating trauma and traumatic grief in children and adolescents* (Second edition). Guilford Press.

[CR10] Complex trauma Treatment Network of the National Child Traumatic Stress Network. (2016). Complex trauma. *Juvenile justice System-Involved youth*. National Center for. https://www.nctsn.org/sites/default/files/resources//complex_trauma_in_juvenile_justice_system_involved_youth.pdf Child Traumatic Stress.

[CR11] Conners, C. K. (2008). Conners third edition (Conners 3). *Los Angeles, CA: Western Psychological Services*.

[CR12] D’Amico, P. J., Vogel, J. M., Mannarino, A. P., Hoffman, D. L., Briggs, E. C., Tunno, A. M., Smith, C. C., Hoover, D., & Schwartz, R. M. (2022). Tailoring Trauma-Focused cognitive behavioral therapy (TF-CBT) for youth with intellectual and developmental disabilities: A survey of nationally certified TF- CBT therapists. *Evidence-Based Practice in Child and Adolescent Mental Health*, *7*(1), 112–124. 10.1080/23794925.2021.1955639

[CR13] Development Service Group, I. (2017). *Youths with intellectual and developmental disabilities in the juvenile justice system*. Office of Juvenile Justice and Delinquency Prevention.

[CR14] Duron, J. F., Williams-Butler, A., Mattson, P., & Boxer, P. (2022). Trauma exposure and mental health needs among adolescents involved with the juvenile justice system. *Journal of Interpersonal Violence*, *37*(17–18), NP15700–NP15725. 10.1177/08862605211016358 34039047 10.1177/08862605211016358

[CR15] Eme, R. F. (2008). Attention-Deficit/Hyperactivity disorder and the juvenile justice system. *Journal of Forensic Psychology Practice*, *8*(2), 174–185. 10.1080/15228930801963994

[CR16] Ergun, G., Schultz, M. S., & Rettig, E. K. (2021). Fetal alcohol spectrum disorder—Issues of misdiagnosis and missed diagnosis in black youth: A case report. *Innovations in Clinical Neuroscience*, *18*(4–6), 20. 34980979 PMC8667706

[CR17] Fabelo, T., Thompson, M. D., Plotkin, M., Carmichael, D., Marchbanks, M. P., & Booth, E. A. (2011). *Breaking schools’ rules: A statewide study of how school discipline relates to students’ success and juvenile justice involvement*. Council of State Governments Justice Center.

[CR18] Fast, D. K., Conry, J., & Loock, C. A. (1999). Identifying fetal alcohol syndrome among youth in the criminal justice system. *Journal of Developmental & Behavioral Pediatrics*, *20*(5). https://journals.lww.com/jrnldbp/fulltext/1999/10000/identifying_fetal_alcohol_syndrome_among_youth_in.12.aspx 10.1097/00004703-199910000-00012 10533996

[CR19] Foa, E. B., Ehlers, A., Clark, D. M., Tolin, D. F., & Orsillo, S. M. (1999). The posttraumatic cognitions inventory (PTCI): Development and validation. *Psychological Assessment*, *11*(3), 303–314. 10.1037/1040-3590.11.3.303

[CR20] Foa, E., Chrestman, K., & Gilboa-Schechtman, E. (2008). *Prolonged exposure manual for children and adolescents suffering from PTSD*.

[CR21] Folk, J. B., Kemp, K., Yurasek, A., Barr-Walker, J., & Tolou-Shams, M. (2021). Adverse childhood experiences among justice-involved youth: Data-driven recommendations for action using the sequential intercept model. *American Psychologist*, *76*(2), 268–283. 10.1037/amp0000769 33734794 10.1037/amp0000769 PMC8281579

[CR22] Folk, J. B., Ramaiya, M., Holloway, E., Ramos, L., Marshall, B. D. L., Kemp, K., Li, Y., Bath, E., Mitchell, D. K., & Tolou-Shams, M. (2022). The association between expanded aces and behavioral health outcomes among youth at first time legal system contact. *Research on Child and Adolescent Psychopathology*. 10.1007/s10802-022-01009-w 36565372 10.1007/s10802-022-01009-w PMC10290175

[CR23] Ford, J. D. (2020). *Trauma affect regulation: Guide for education and therapy.*

[CR25] Ford, J. D., Steinberg, K. L., Hawke, J., Levine, J., & Zhang, W. (2012). Randomized trial comparison of emotion regulation and relational psychotherapies for PTSD with girls involved in delinquency. *Journal of Clinical Child & Adolescent Psychology*, *41*(1), 27–37. 10.1080/15374416.2012.632343 22233243 10.1080/15374416.2012.632343

[CR24] Ford, J. D., Grasso, D. J., Hawke, J., & Chapman, J. F. (2013). Poly-victimization among juvenile justice-involved youths. *Child Abuse & Neglect*, *37*(10), 788–800. 10.1016/j.chiabu.2013.01.005 23428165 10.1016/j.chiabu.2013.01.005

[CR26] Greenwald, R. (2000). A Trauma-Focused individual therapy approach for adolescents with conduct disorder. *International Journal of Offender Therapy and Comparative Criminology*, *44*(2), 146–163. 10.1177/0306624X00442002

[CR27] Grigorenko, E. L., Macomber, D., Hart, L., Naples, A., Chapman, J., Geib, C. F., Chart, H., Tan, M., Wolhendler, B., & Wagner, R. (2015). Academic achievement among juvenile detainees. *Journal of Learning Disabilities*, *48*(4), 359–368. 10.1177/0022219413500991 24064502 10.1177/0022219413500991 PMC5064284

[CR28] Heilbrun, K., Goldstein, N. E. S., DeMatteo, D., Newsham, R., Gale-Bentz, E., Cole, L., & Arnold, S. (2017). The sequential intercept model and juvenile justice: Review and prospectus. *Behavioral Sciences & the Law*, *35*(4), 319–336. 10.1002/bsl.2291 28612513 10.1002/bsl.2291

[CR29] Jaycox, L. H., Langley, A. K., & Hoover, S. A. (2018). *Cognitive behavioral intervention for trauma in schools (CBITS): Second edition*. RAND Corporation. https://www.rand.org/pubs/tools/TL272.html

[CR30] Johnson, W. F. L., & Davis, D. ( 2020). *Cognitive Behavioral Interventions for Trauma in Schools (CBITS) with Cultural Adaptations (CBITS-CA) Expansion at Echo Glen Children’s Center*. 48.

[CR31] Kaplow, J. B., Rolon-Arroyo, B., Layne, C. M., Rooney, E., Oosterhoff, B., Hill, R., Steinberg, A. M., Lotterman, J., Gallagher, K. A. S., & Pynoos, R. S. (2020). Validation of the UCLA PTSD reaction index for DSM-5: A developmentally informed assessment tool for youth. *Journal of the American Academy of Child & Adolescent Psychiatry*, *59*(1), 186–194. 10.1016/j.jaac.2018.10.019 30953734 10.1016/j.jaac.2018.10.019

[CR32] Keesler, J. M. (2020). Trauma-Specific treatment for individuals with intellectual and developmental disabilities: A review of the literature from 2008 to 2018. *Journal of Policy and Practice in Intellectual Disabilities*, *17*(4), 332–345. 10.1111/jppi.12347

[CR33] Kim, B. K. E., Johnson, J., Rhinehart, L., Logan-Greene, P., Lomeli, J., & Nurius, P. S. (2021). The school-to-prison pipeline for probation youth with special education needs. *American Journal of Orthopsychiatry*, *91*(3), 375–385. 10.1037/ort0000538 34138628 10.1037/ort0000538 PMC8432608

[CR34] Kvarfordt, C. L., Purcell, P., & Shannon, P. (2005). Youth with learning disabilities in the juvenile justice system: A training needs assessment of detention and court services personnel. *Child and Youth Care Forum*, *34*(1), 27–42. 10.1007/s10566-004-0880-x

[CR35] Legha, R. K. (2025). There are no bad kids: An antiracist approach to oppositional defiant disorder. *Pediatrics*, *155*(2), e2024068415. 10.1542/peds.2024-068415 39786560 10.1542/peds.2024-068415

[CR36] Mayer, B., Elbing, U., & Ostermann, T. (2023). Trauma treatment using narrative exposure therapy adapted to persons with intellectual disabilities or severe chronic mental disorders – a randomised controlled pilot study with an embedded observational study. *Journal of Intellectual Disability Research*, *67*(11), 1096–1112. 10.1111/jir.13076 37582663 10.1111/jir.13076

[CR37] Mendel, R. A. (2011). *No place for kids: The case for reducing juvenile incarceration*.

[CR38] Merten, E. C., Cwik, J. C., Margraf, J., & Schneider, S. (2017). Overdiagnosis of mental disorders in children and adolescents (in developed countries). *Child and Adolescent Psychiatry and Mental Health*, *11*(1), 5. 10.1186/s13034-016-0140-5 28105068 10.1186/s13034-016-0140-5 PMC5240230

[CR39] Morgan, P. L., Woods, A. D., & Wang, Y. (2023). Sociodemographic disparities in attention-deficit/hyperactivity disorder overdiagnosis and overtreatment during elementary school. *Journal of Learning Disabilities*, *56*(5), 359–370. 10.1177/00222194221099675 35674454 10.1177/00222194221099675 PMC10426255

[CR40] Morris, K. A., & Morris, R. J. (2006). Disability and juvenile delinquency: Issues and trends. *Disability & Society*, *21*(6), 613–627. 10.1080/09687590600918339

[CR41] National Center for PTSD, U.S. Department of Veterans Affairs (2025). *Complex PTSD: History and Definitions*. PTSD: National Center for PTSD. https://www.ptsd.va.gov/professional/treat/essentials/complex_ptsd.asp

[CR42] National Council on Disability (2015). *Breaking the School-to-Prison Pipeline for Students with Disabilities*. https://www.ncd.gov/report/breaking-the-school-to-prison-pipeline-for-students-with-disabilities/

[CR43] Newman, E. (2002). Assessment of PTSD and trauma exposure in adolescents. *Journal of Aggression Maltreatment & Trauma*, *6*(1), 59–77. 10.1300/J146v06n01_04

[CR44] Ochoa, T. A., Datchi, C. C., Weller, N. M., Bohmert, N., M., & Grubbs, D. (2021). Education and transition for students with disabilities in American juvenile correctional facilities. *Intervention in School and Clinic*, *56*(5), 293–300. 10.1177/1053451220963089

[CR45] Reinecke, M. A., & Shirk, S. R. (2007). Psychotherapy with adolescents. In G. O. Gabbard, J. S. Beck, & J. Holmes (Eds.), *Oxford textbook of psychotherapy* (Reprinted). Oxford Univ. Press.

[CR46] Resick, P. A., & Schnicke, M. (1993). *Cognitive processing therapy for rape victims: A treatment manual*. Sage.

[CR58] Rhoden, M.-A., Macgowan, M. J., & Huang, H. (2019). A systematic review of psychological trauma interventions for juvenile offenders. *Research on Social Work Practice*, *29*(8), 892–909. 10.1177/104973151880657

[CR47] Rodriguez, N. (2013). Concentrated disadvantage and the incarceration of youth: Examining how context affects juvenile justice. *Journal of Research in Crime and Delinquency*, *50*(2), 189–215. 10.1177/0022427811425538

[CR48] Saltzman, W. (2017). *Trauma and grief component therapy for adolescents: A modular approach to treating traumatized and bereaved youth*. Cambridge University Press.

[CR49] Schramm, A. T., Pandya, K., Fairchild, A. J., Venta, A. C., deRoon-Cassini, T. A., & Sharp, C. (2020). Decreases in psychological inflexibility predict PTSD symptom improvement in inpatient adolescents. *Journal of Contextual Behavioral Science*, *17*, 102–108. 10.1016/j.jcbs.2020.06.007

[CR50] Stone, S., & Zibulsky, J. (2015). Maltreatment, academic difficulty, and systems-involved youth: Current evidence and opportunities. *Psychology in the Schools*, *52*(1), 22–39. 10.1002/pits.21812

[CR51] Streissguth, A. P., Barr, H. M., Kagan, J., & Bookstein, F. L. (1996). *Understanding the Occurrence of Secondary Disabilities in Clients with Fetal Alcohol Syndrome (FAS) and Fetal Alcohol Effects (FAE)* (Centers for Disease Control and Prevention Grant No. R04/CCR008515). University of Washington School of Medicine Department of Psychiatry and Behavioral Sciences Fetal Alcohol and Drug Unit. http://lib.adai.uw.edu/pubs/bk2698.pdf

[CR52] Sukhodolsky, D. G., & Ruchkin, V. (2006). Evidence-Based psychosocial treatments in the juvenile justice system. *Child and Adolescent Psychiatric Clinics*, *15*(2), 501–516. 10.1016/j.chc.2005.11.005 10.1016/j.chc.2005.11.005 16527668

[CR53] Sweeten, G. (2006). Who will graduate? Disruption of high school education by arrest and court involvement. *Justice Quarterly*, *23*(4), 462–480. 10.1080/07418820600985313

[CR54] Vogel, A., & Rosner, R. (2020). Lost in transition? Evidence-based treatments for adolescents and young adults with posttraumatic stress disorder and results of an uncontrolled feasibility trial evaluating cognitive processing therapy. *Clinical Child and Family Psychology Review*, *23*(1), 122–152. 10.1007/s10567-019-00305-0 31620891 10.1007/s10567-019-00305-0

[CR55] Wang, X., Blomberg, T. G., & Li, S. D. (2005). Comparison of the educational deficiencies of delinquent and nondelinquent students. *Evaluation Review*, *29*(4), 291–312. 10.1177/0193841X05275389 15985521 10.1177/0193841X05275389

[CR56] Zettler, H. R. (2021). Much to do about trauma: A systematic review of existing trauma-informed treatments on youth violence and recidivism. *Youth Violence and Juvenile Justice*, *19*(1), 113–134. 10.1177/1541204020939645

[CR57] Zhang, D., Hsu, H. Y., Katsiyannis, A., Barrett, D. E., & Ju, S. (2011). Adolescents with disabilities in the juvenile justice system: Patterns of recidivism. *Exceptional Children*, *77*(3), 283–298. 10.1177/001440291107700302

