# Supplementary material for: A Scoping Review of Trauma Treatments among Legally Involved Adolescents with Learning, Cognitive, or Intellectual Disabilities: Identifying Clinically Relevant Research Gaps in the Literature
Source: J Child Adolesc Trauma. 2025 Aug 18;19(1):209–21. doi: 10.1007/s40653-025-00742-w (PMC12889888; doi:10.1007/s40653-025-00742-w)
Supplement: Supplementary file 1 — Supplementary Material 1 [file 40653_2025_742_MOESM1_ESM.docx]

Supplemental Material A. Search strategies for all databases. All searches were conducted on May 1, 2023. Date limits of 1958-2023 were used.

| Database | Search Strategy | Number of Results |
| --- | --- | --- |
| PubMed (1966-) | "Therapeutics/psychology"[MeSH] OR counsel*[tiab] OR debrief*[tiab] OR "psychological first aid"[tiab] OR "group therapy"[tiab] OR "TF-CBT"[tiab] OR "tf cbt"[tiab] OR "trauma-focused cognitive behavioral therapy"[tiab] OR "trauma focused cbt"[tiab] OR "cognitive behavioral therapy"[tiab] OR cbt[tiab] OR emdr[tiab] OR “Eye movement desensitization”[tiab] OR “Eye movement desensitization and reprocessing”[tiab] OR cpt[tiab] OR "cognitive processing therapy"[tiab] OR "exposure therapy"[tiab] OR "seeking safety"[tiab] OR cbits[tiab] OR "cognitive behavioral intervention for trauma in schools"[tiab] OR TGCTA[tiab] OR "sanctuary model"[tiab] OR fft[tiab] OR "functional family therapy"[tiab] OR mst[tiab] OR "multisystemic therapy"[tiab] OR pe[tiab] OR "prolonged exposure"[tiab] OR "pe-a"[tiab] OR "adolescent psychotherapy"[tiab]  AND  “psychological trauma”[MeSH] OR "Stress Disorders, Post-Traumatic"[Mesh] OR “trauma and stressor related disorders”[MeSH] OR "Stress Disorders, Traumatic"[MeSH] OR “adverse childhood experiences”[MeSH] OR “childhood adversity”[tiab] OR “adverse childhood event”[tiab] OR “child abuse”[MeSH] OR "posttraumatic stress disorder" OR "ptsd"  AND  "Juvenile Delinquency"[Mesh] OR incarcerat*[tiab] OR probation[tiab] OR “community supervised”[tiab] OR arrest[tiab] OR arrested[tiab] OR detention[tiab] OR detained[tiab] OR jail[tiab] OR jailed[tiab] OR jails[tiab] OR imprisoned[tiab] OR corrections[tiab] OR correctional[tiab] OR offender[tiab] OR offenders[tiab] OR reoffending[tiab] OR justice[tiab] OR justiceinvolved[tiab] OR justice involved"[tiab] OR diversion[tiab] OR diverted[tiab] OR "Juvenile Delinquency"[Mesh] OR court-involved[tiab] OR "juvenile court"[tiab] OR "family court"[tiab] OR “cross-over”[tiab] OR crossover[tiab] OR "training school"[tiab] OR "group home"[tiab] OR "residential placement"[tiab] OR "legal involvement"[tiab] OR "legally involved"[tiab] OR delinquent[tiab] OR "status offenses"[tiab] OR truan*[tiab] OR absente*[tiab] OR adjudicat*[tiab]  AND  adolescent[MeSH] OR child[MeSH] OR juvenile[tiab] or minor[tiab]  AND  “Learning Disabilities”[Mesh] OR "Specific language Disorder"[Mesh] OR "Developmental Disabilities"[MeSH] OR "Neurodevelopmental Disorders"[Mesh] OR "Intellectual Disability"[MeSH] OR "Attention Deficit Disorder with Hyperactivity"[MeSH] OR "Autism Spectrum Disorder"[Mesh] OR "developmental delay"[tiab] | 4 |
| PsycINFO (ProQuest, 1887-) | MAINSUBJECT.EXACT("Eye Movement Desensitization Therapy") OR MAINSUBJECT.EXACT.EXPLODE("Adolescent Psychotherapy") OR MAINSUBJECT.EXACT.EXPLODE("Cognitive Behavior Therapy") OR MAINSUBJECT.EXACT.EXPLODE("Exposure Therapy") OR MAINSUBJECT.EXACT.EXPLODE("Psychotherapy") OR MAINSUBJECT.EXACT("Multisystemic Therapy") OR MAINSUBJECT.EXACT("Cognitive Processing Therapy") OR MAINSUBJECT.EXACT.EXPLODE("School Based Intervention") OR MAINSUBJECT.EXACT.EXPLODE("Group Intervention") OR MAINSUBJECT.EXACT.EXPLODE("Response to Intervention") OR MAINSUBJECT.EXACT.EXPLODE("Family Intervention") OR MAINSUBJECT.EXACT.EXPLODE("Intervention") OR MAINSUBJECT.EXACT.EXPLODE("Debriefing (Psychological)") OR counsel* OR debrief* OR "psychological first aid" OR "group therapy" OR "TF-CBT" OR "tf cbt" OR "trauma-focused cognitive behavioral therapy" OR "trauma focused cbt" OR "seeking safety" OR “Trauma Affect Regulation: A Guide for Education and Therapy” OR sitcap OR "Structured sensory intervention for traumatized children, adolescents, and parents” OR "SITCAP-ART" OR “Structured sensory intervention for traumatized children, adolescents, and parent—adjudicated and at-risk Youth” OR cbits OR "cognitive behavioral intervention for trauma in schools" OR TGCTA OR “Trauma and Grief Component Therapy for Adolescents” OR "think trauma" OR "sanctuary model" OR fft OR "functional family therapy" OR mst OR "multisystemic therapy" OR "pe-a" OR "prolonged exposure for adolescents"  AND  MAINSUBJECT.EXACT.EXPLODE("Stress and Trauma Related Disorders") OR MAINSUBJECT.EXACT.EXPLODE("Trauma") OR MAINSUBJECT.EXACT("Posttraumatic Stress") OR MAINSUBJECT.EXACT.EXPLODE("adversity") OR MAINSUBJECT.EXACT("Child Abuse") OR “adverse childhood experiences” OR “childhood adversity” OR “adverse childhood event”  AND  MAINSUBJECT.EXACT.EXPLODE("Truancy") OR MAINSUBJECT.EXACT("Juvenile Delinquency") OR MAINSUBJECT.EXACT.EXPLODE("Law Enforcement") OR MAINSUBJECT.EXACT.EXPLODE("Legal Processes") OR MAINSUBJECT.EXACT.EXPLODE("Adjudication") OR MAINSUBJECT.EXACT("Child Welfare") OR MAINSUBJECT.EXACT.EXPLODE("Juvenile Justice") OR MAINSUBJECT.EXACT("Juvenile Justice") OR MAINSUBJECT.EXACT("Legal Arrest") OR MAINSUBJECT.EXACT.EXPLODE("Diversion Programs") OR MAINSUBJECT.EXACT.EXPLODE("Juvenile Delinquency") OR MAINSUBJECT.EXACT.EXPLODE("Justice") OR incarcerat* OR probation OR “community supervised” OR detention OR detained OR jail OR jailed OR jails OR imprisoned OR corrections OR correctional OR offender OR offenders OR reoffending OR justice OR “justice-involved” OR “justice involved" OR “court-involved” OR "juvenile court" OR "family court" OR cross-over OR crossover OR "training school" OR "group home" OR "residential placement" OR "legal involvement" OR "legally involved" OR diverted OR diversion OR delinquent OR "status offenses" OR absente*  AND  adolescent OR child OR juvenile OR minor  AND  MAINSUBJECT.EXACT.EXPLODE("Neurodevelopmental Disorders") OR MAINSUBJECT.EXACT.EXPLODE("Intellectual Development Disorder") OR MAINSUBJECT.EXACT.EXPLODE("Autism Spectrum Disorders") OR MAINSUBJECT.EXACT.EXPLODE("Intellectual Development") OR MAINSUBJECT.EXACT.EXPLODE("Learning Disabilities") OR MAINSUBJECT.EXACT.EXPLODE("Developmental Disabilities") OR MAINSUBJECT.EXACT.EXPLODE("Disabilities") OR "Specific language Disorder" OR "Intellectual Disability" OR "Attention Deficit Disorder with Hyperactivity" OR "developmental delay" OR Dyscalculia OR Dyslexia OR "attention deficit disorder" OR "attention deficit hyperactivity disorder" | 35 |
| Social Services Abstracts (ProQuest, 1963- and 1980- ) | MAINSUBJECT.EXACT("Intervention") OR MAINSUBJECT.EXACT.EXPLODE("Treatment") OR MAINSUBJECT.EXACT.EXPLODE("Treatment Methods") OR MAINSUBJECT.EXACT.EXPLODE("Treatment Outcomes") OR MAINSUBJECT.EXACT.EXPLODE("Psychotherapy") OR MAINSUBJECT.EXACT.EXPLODE("Counseling") OR MAINSUBJECT.EXACT.EXPLODE("Treatment Programs") OR MAINSUBJECT.EXACT.EXPLODE("Group Therapy") OR MAINSUBJECT.EXACT.EXPLODE("Counseling") OR debrief* OR "psychological first aid" OR "group therapy" OR "TF-CBT" OR "tf cbt" OR "trauma-focused cognitive behavioral therapy" OR "trauma focused cbt" OR "cognitive behavioral therapy" OR cbt OR emdr OR “Eye movement desensitization” OR “Eye movement desensitization and reprocessing” OR cpt OR "cognitive processing therapy" OR "exposure therapy" OR "seeking safety" OR “Trauma Affect Regulation: A Guide for Education and Therapy” OR sitcap OR "Structured sensory intervention for traumatized children, adolescents, and parents” OR "SITCAP-ART" OR “Structured sensory intervention for traumatized children, adolescents, and parent—adjudicated and at-risk Youth” OR cbits OR "cognitive behavioral intervention for trauma in schools" OR TGCTA OR “Trauma and Grief Component Therapy for Adolescents” OR "think trauma" OR "sanctuary model" OR fft OR "functional family therapy" OR mst OR "multisystemic therapy" OR pe OR "prolonged exposure" OR "pe-a" OR "prolonged exposure for adolescents"  AND  MAINSUBJECT.EXACT.EXPLODE("Posttraumatic Stress Disorder") OR MAINSUBJECT.EXACT.EXPLODE("Child Abuse") OR MAINSUBJECT.EXACT.EXPLODE("Trauma") OR “psychological trauma” OR “trauma and stressor related disorders” OR "Stress Disorders, Traumatic" OR “adverse childhood experiences” OR “childhood adversity” OR “adverse childhood event” OR “child abuse”  AND  MAINSUBJECT.EXACT.EXPLODE("Juvenile Justice") OR MAINSUBJECT.EXACT.EXPLODE("Juvenile Correctional Institutions") OR MAINSUBJECT.EXACT.EXPLODE("Probation") OR MAINSUBJECT.EXACT.EXPLODE("Arrests") OR MAINSUBJECT.EXACT.EXPLODE("Absenteeism") OR MAINSUBJECT.EXACT.EXPLODE("Correctional System") OR MAINSUBJECT.EXACT.EXPLODE("Imprisonment") OR MAINSUBJECT.EXACT.EXPLODE("Juvenile Delinquency") OR MAINSUBJECT.EXACT.EXPLODE("Child Welfare Services") OR MAINSUBJECT.EXACT.EXPLODE("Detention") OR MAINSUBJECT.EXACT.EXPLODE("Delinquency Prevention") OR MAINSUBJECT.EXACT.EXPLODE("Offenders") OR MAINSUBJECT.EXACT.EXPLODE("Juvenile Offenders") OR MAINSUBJECT.EXACT.EXPLODE("Juvenile Courts") OR MAINSUBJECT.EXACT.EXPLODE("Truancy") OR incarcerat* OR “community supervised” OR arrest OR arrested OR detention OR detained OR jail OR jailed OR jails OR imprisoned OR corrections OR correctional OR offender OR offenders OR reoffending OR justice OR “justice-involved” OR “justice involved" OR diversion OR diverted OR “court-involved” OR "juvenile court" OR "family court" OR cross-over OR crossover OR "training school" OR "group home" OR "residential placement" OR "legal involvement" OR "legally involved" OR delinquent OR "status offenses"  AND  MAINSUBJECT.EXACT.EXPLODE("Adolescents") OR MAINSUBJECT.EXACT("Young Adults") OR MAINSUBJECT.EXACT("Children") OR MAINSUBJECT.EXACT("Youth")  AND  MAINSUBJECT.EXACT.EXPLODE("Autism") OR MAINSUBJECT.EXACT.EXPLODE("Learning Disabilities") OR MAINSUBJECT.EXACT.EXPLODE("Developmental Disabilities") OR MAINSUBJECT.EXACT.EXPLODE("Language Disorders") OR MAINSUBJECT.EXACT.EXPLODE("Attention Deficit Disorder") OR "neurodevelopmental disabilities" OR "neurodevelopmental disability" OR "Intellectual Development" OR "autism spectrum disorder" OR "attention deficit hyperactivity disorder" OR "global developmental delay" or "developmental delay" OR "dyscalculia" OR "dyslexia" | 123 |
| PTSDPubs (1871-) | MAINSUBJECT.EXACT.EXPLODE("Trauma Focused Cognitive Behavioral Therapy") OR MAINSUBJECT.EXACT.EXPLODE("Critical Incident Stress Debriefing") OR MAINSUBJECT.EXACT.EXPLODE("Cognitive Behavioral Therapy") OR MAINSUBJECT.EXACT.EXPLODE("Psychological Debriefing") OR MAINSUBJECT.EXACT.EXPLODE("EMDR") OR MAINSUBJECT.EXACT.EXPLODE("Group Psychotherapy") OR MAINSUBJECT.EXACT.EXPLODE("Exposure Therapy") OR MAINSUBJECT.EXACT.EXPLODE("Trauma Focused Group Psychotherapy") OR MAINSUBJECT.EXACT.EXPLODE("Prolonged Exposure Therapy") OR MAINSUBJECT.EXACT.EXPLODE("Cognitive Processing Therapy") OR counsel* OR "psychological first aid" OR "seeking safety" OR “Trauma Affect Regulation: A Guide for Education and Therapy” OR sitcap OR "Structured sensory intervention for traumatized children, adolescents, and parents” OR "SITCAP-ART" OR “Structured sensory intervention for traumatized children, adolescents, and parent—adjudicated and at-risk Youth” OR cbits OR "cognitive behavioral intervention for trauma in schools" OR TGCTA OR “Trauma and Grief Component Therapy for Adolescents” OR "think trauma" OR "sanctuary model" OR fft OR "functional family therapy" OR mst OR "multisystemic therapy" OR "pe-a" OR "prolonged exposure for adolescents"  AND  MAINSUBJECT.EXACT.EXPLODE("Criminal Behavior") OR MAINSUBJECT.EXACT.EXPLODE("Incarceration") OR incarcerat* OR probation OR “community supervised” OR arrest OR arrested OR detention OR detained OR jail OR jailed OR jails OR imprisoned OR corrections OR correctional OR offender OR offenders OR reoffending OR justice OR “justice-involved” OR “justice involved" OR diversion OR diverted OR "Juvenile Delinquency" OR “court-involved” OR "juvenile court" OR "family court" OR cross-over OR crossover OR "training school" OR "group home" OR "residential placement" OR "legal involvement" OR "legally involved" OR diverted OR diversion OR delinquent OR "status offenses" OR truan* OR absente*  AND  MAINSUBJECT.EXACT.EXPLODE("Adolescents") OR MAINSUBJECT.EXACT.EXPLODE("Children") OR MAINSUBJECT.EXACT.EXPLODE("Young Adults") OR juvenile OR minor  AND  MAINSUBJECT.EXACT.EXPLODE("Language Disorders") OR MAINSUBJECT.EXACT.EXPLODE("ADHD") OR MAINSUBJECT.EXACT.EXPLODE("Mentally Retarded") OR MAINSUBJECT.EXACT.EXPLODE("Cognitive Impairment") OR MAINSUBJECT.EXACT.EXPLODE("Pervasive Developmental Disorders") OR “Learning disabilities” OR Dyscalculia OR Dyslexia OR "specific learning disorder" OR "developmental disabilities" OR "neurodevelopmental disorders" OR "intellectual disability" OR "Attention Deficit Disorder with Hyperactivity" OR "Autism Spectrum Disorder" OR "developmental delay” OR "global developmental delay" OR "attention deficit disorder" | 2 |
| Embase (1947-) | psychotherapy'/exp OR 'counseling'/exp OR 'therapy effect'/exp OR 'eye movement desensitization and reprocessing'/exp OR 'cognitive behavioral therapy'/exp OR 'cognitive processing therapy'/exp OR 'trauma-focused cognitive behavioral therapy'/exp OR 'group therapy'/exp OR treatment OR intervention OR debrief* OR “psychological first aid” OR “Eye movement desensitization and reprocessing” OR “exposure therapy” OR “seeking safety” OR “Trauma Affect Regulation: A Guide for Education and Therapy” OR sitcap OR “Structured sensory intervention for traumatized children, adolescents, and parents” OR “SITCAP-ART” OR “Structured sensory intervention for traumatized children, adolescents, and parent—adjudicated and at-risk Youth” OR cbits OR “cognitive behavioral intervention for trauma in schools” OR TGCTA OR “Trauma and Grief Component Therapy for Adolescents” OR “think trauma” OR “sanctuary model” OR fft OR “functional family therapy” OR mst OR “multisystemic therapy” OR “pe-a” OR “prolonged exposure for adolescents”  AND  psychotrauma'/exp OR 'posttraumatic stress disorder'/exp OR 'acute stress disorder'/exp OR 'childhood adversity'/exp OR “psychological trauma” OR “trauma and stressor related disorders” OR "Traumatic stress disorder" OR “adverse childhood experiences” OR “adverse childhood event” OR “child abuse”  AND  juvenile delinquency'/exp OR 'prisoner'/exp OR 'correctional facility'/exp OR 'truancy'/exp OR incarcerated OR incarceration OR probation OR “community supervised” OR arrest OR arrested OR detention OR detained OR jail OR jailed OR jails OR imprisoned OR corrections OR correctional OR offender OR offenders OR reoffending OR justice OR “justice-involved” OR “justice involved" OR diversion OR diverted OR “court-involved” OR "juvenile court" OR "family court" OR cross-over OR crossover OR "training school" OR "group home" OR "residential placement" OR "legal involvement" OR "legally involved" OR diverted OR diversion OR delinquent OR "status offenses" OR absentee OR absenteeism  AND  child'/exp OR 'adolescent'/exp OR 'minor (person)'/exp OR 'young adult'/exp OR 'adolescence'/exp OR teenage  AND  learning disorder'/exp OR 'language disability'/exp OR 'intellectual impairment'/exp OR 'developmental delay'/exp OR 'developmental disorder'/exp OR 'autism'/exp OR 'attention deficit hyperactivity disorder'/exp OR 'dyscalculia'/exp OR 'dyslexia'/exp OR "global developmental delay" OR "attention deficit disorder" OR "neurodevelopmental disorders" | 147 |
| Web of Science (1900-) | TS=("Therapeutics" OR therapy * OR treatment OR intervention OR counsel* OR debrief* OR "psychological first aid" OR "group therapy" OR "TF-CBT" OR "tf cbt" OR "trauma-focused cognitive behavioral therapy" OR "trauma focused cbt" OR "cognitive behavioral therapy" OR cbt OR emdr OR “Eye movement desensitization” OR “Eye movement desensitization and reprocessing” OR cpt OR "cognitive processing therapy" OR "exposure therapy" OR "seeking safety" OR “Trauma Affect Regulation: A Guide for Education and Therapy” OR sitcap OR "Structured sensory intervention for traumatized children, adolescents, and parents” OR "SITCAP-ART" OR “Structured sensory intervention for traumatized children, adolescents, and parent—adjudicated and at-risk Youth” OR cbits OR "cognitive behavioral intervention for trauma in schools" OR TGCTA OR “Trauma and Grief Component Therapy for Adolescents” OR "think trauma" OR "sanctuary model" OR fft OR "functional family therapy" OR mst OR "multisystemic therapy" OR pe OR "prolonged exposure" OR "pe-a" OR "prolonged exposure for adolescents")  AND  TS=(“psychological trauma” OR "posttraumatic stress disorder" OR “trauma and stressor related disorders” OR "Traumatic stress disorder" OR “adverse childhood experiences” OR “childhood adversity” OR “adverse childhood event” OR “child abuse”)  AND  ALL=(incarcerat* OR probation OR “community supervised” OR arrest OR arrested OR detention OR detained OR jail OR jailed OR jails OR imprisoned OR corrections OR correctional OR offender OR offenders OR reoffending OR justice OR “justice-involved” OR “justice involved" OR diversion OR diverted OR "Juvenile Delinquency" OR “court-involved” OR "juvenile court" OR "family court" OR cross-over OR crossover OR "training school" OR "group home" OR "residential placement" OR "legal involvement" OR "legally involved" OR diverted OR diversion OR delinquent OR "status offenses" OR truan* OR absente*)  AND  TS=(adolescent OR "young adult" OR child OR juvenile OR minor OR teenag*)  AND  ALL=(“Learning disabilities” OR Dyscalculia OR Dyslexia OR "specific learning disorder" OR "specific language disorder" OR "developmental disabilities" OR "neurodevelopmental disorders" OR "intellectual disability" OR "Attention Deficit Disorder with Hyperactivity" OR "Autism Spectrum Disorder" OR "developmental delay” OR "global developmental delay" OR "attention deficit disorder") | 18 |
| Google Scholar | ("juvenile justice" OR "court-involved youth") AND (trauma OR "posttraumatic stress") AND (therapy OR treatment) AND ("learning disabilities" OR "developmental disabilities") | 50 |
| Total number of results from database search | | 379 |
| Total number of duplicates | | 17 |
| Total results after de-duplication | | 362 |
